# Data Set For Computation Of Maxillary Arch Perimeter With Ramanujan's Equation For Ellipse In Different Skeletal Malocclusions.

**DOI:** 10.1016/j.dib.2020.106079

**Published:** 2020-07-25

**Authors:** Gowri Sankar Singaraju, Yamini Priyanka JS, Prasad Mandava, Vivek Reddy Ganugapanta, Naga Ravi Teja, Praveen Reddy JN

**Affiliations:** a Department of Orthodontics, Narayana Dental College and Hospital, Nellore, Andhra Pradesh, India-524003; b YourDOST Health Solutions, Bangalore, Karnataka, Andhra Pradesh, India-560095

**Keywords:** Orthodontics, ARCH perimeter, TSALD, Ellipse, Ramanujan's equation, Inter-molar width, Inter-canine width

## Abstract

Every practicing orthodontist today is aware of the importance of considering arch form in the attainment of a functional orthodontic correction [1]. Arch perimeter or circumference prediction is an essential component when Tooth Size Arch Length Discrepancy (TSALD) is estimated. Arch perimeter is the distance from mesial contact of the permanent molar on one side to the mesial contact of the permanent molar on the other side, with the line connecting the buccal/incisor tip points in the intervening teeth. This is most evident when seeking to resolve dental crowding or arch-length discrepancy (ALD) [2]. The shape of the arch form of maxillary and mandible resembles that of the various geometric forms such as including ellipse, parabola, hyperbola, and catenary curve [3], [4], [5], [6]. Ellipse is the best form that fits the shape of the Maxillary arch [1,2]. The mathematical equation formulated by Srinivasan Ramanujan in 1914 for widely considered to be the most accurate for calculation of the circumference of an ellipse is [7]. The computation of the circumference of the ellipse by this equation requires two values- ‘*a’* and ‘*b,'* the semi-major and semi-minor axis [half of the major axis and minor axis of the ellipse] respectively [8]. The perimeter (P) of an ellipse is given by the formulae; = π(a+b){1+(3h/(10-√(4-3h))}; where h=(a-b)^2^/(a+b)^2^ and calculated Maxillary arch perimeter (CP) =1/2 P. This necessitates a complex series of steps, and to overcome this, a statistical formula is developed by algorithm steps for mathematical equation where perimeter can be directly obtained by just two inputs ’a’ and ’b’ in excel sheet. We correlated this calculated arch perimeter (CP) with directly measured perimeter (MP) and marginal difference estimated in three different classes of malocclusion.

Specifications table

**Table:** 

**Subject**	Medicine and Dentistry (General)
**Specific subject area**	Orthodontics. Diagnosis and Treatment planning
**Type of data**	Table Chart Graph Figure
**How data were acquired**	1. Direct measurement on plaster models with Digital Vernier calipers*(iGaging ®, LA, California, USA)*. 2. A statistical formula for direct data entry of a complex mathematical equation is generated by algorithmic steps.
**Data format**	Raw Analysed
**Parameters for data collection**	A geometric shape of ellipse was fitted to the Maxillary arch. The Inter-molar width (half the minor axis), Inter-canine width and Inter-molar perpendicular (half the major axis) were measured on the model. The arch perimeter was measured. A statistical equation was developed so as the input of the major axis and minor axis generates directly the arch perimeter. The calculated and Measured arch perimeter were compared for correlation
**Description of data collection**	The data collected is basically numerically data on a continuous scale. All the measurements and calculated values are expressed in millimetre(mm).
**Data source location**	Institution: Narayana Dental college City/Town/Region: Nellore, Andhra Pradesh-524003 Country: India Latitude and longitude (and GPS coordinates, if possible) for collected samples/data: 14.4289° N, 80.0120° E
**Data accessibility**	Repository name: **Mendeley Data** Data identification number: 10.17632/ryc5y6c7k7.2 Direct URL to data: https://data.mendeley.com/datasets/ryc5y6c7k7/2

## Value of the data

• The data set can be useful for space calculation of the amount of space available for correction of minor class I, Class II, and class III inter-arch malocclusions.
• The statistical equation generated can simplify the data generation entry for variables involving similar data in different vertical growth patterns and for different population groups
• Standardization of the arch forms can be made based on the ellipse form obtained.
• Clinically, the most suitable and accurate preformed arch wire according to each patient's pre-treatment arch form can be selected
• The relation between the ratio of the Inter canine width and Inter molar width can be established to define the shape of the arch.

## 1 Data description

**Figure 1:**
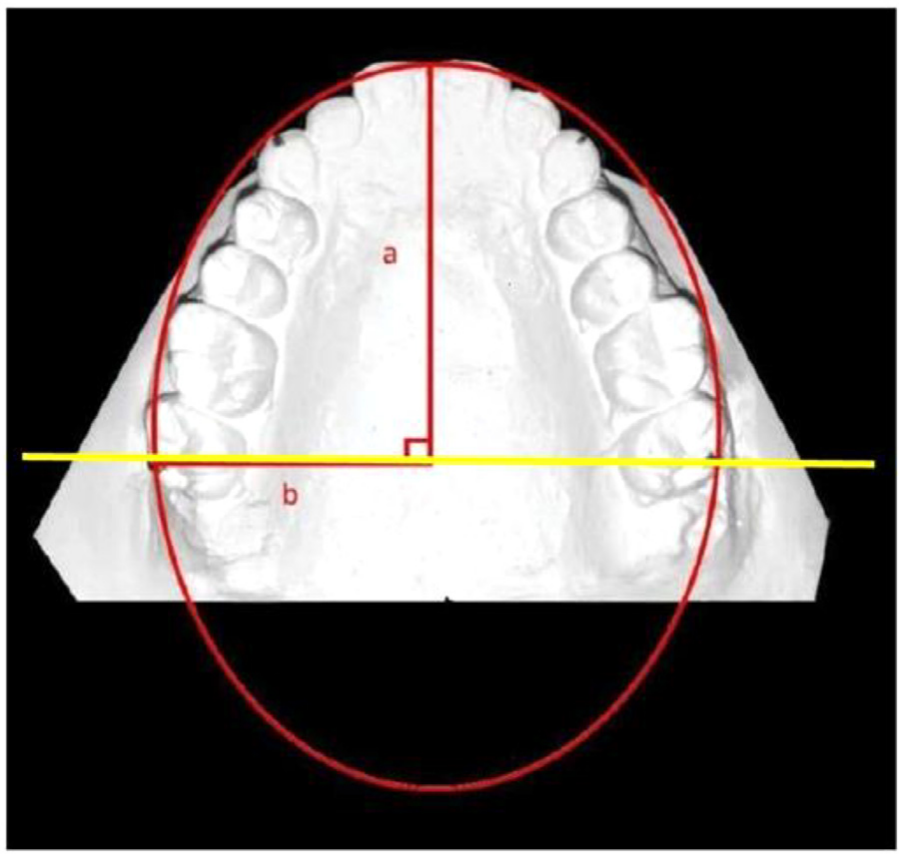
**Ramanujan's equation of Ellipse model**. Fitting of the ellipse for the maxillary arch schematically, where ‘a’ is the semimajor axis, and ‘b’ is the semiminor axis. perimeter of ellipsoid ‘P’= π(a+b){1+(3h/(10+√(4-3h))}; where h=(a-b)^2^/(a+b)^2^. The calculated Maxillary arch perimeter (CP) =1/2 P.

**Figure 2:**
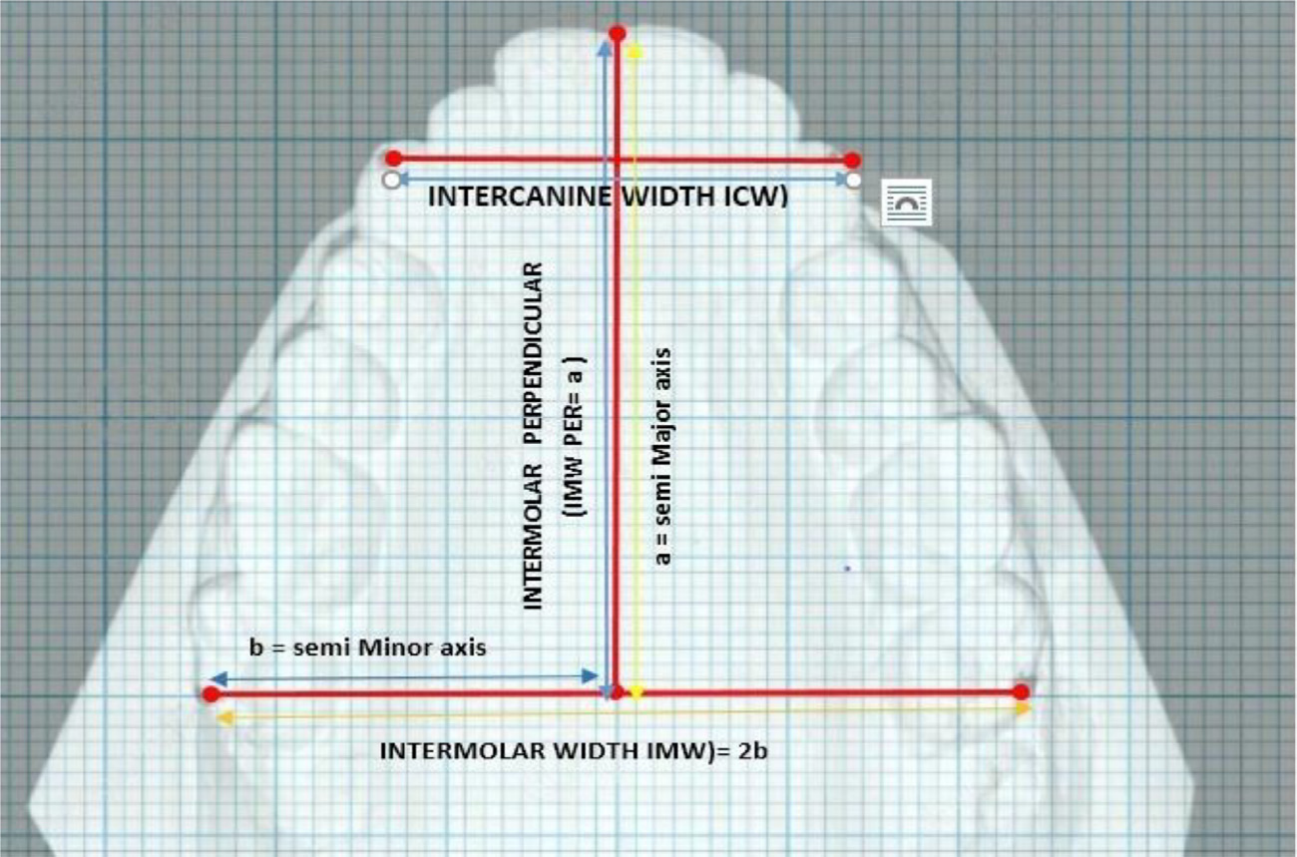
**Parameters defined in the collection of the data. Inter molar width (IMW)=2b**: The distance from the distobuccal cusp of the second molar to contralateral second molar. This is equivalent to twice the semi minor axis of ellipsoid; **Intercanine width (ICW):** The distance from one canine cusp tip to contralateral canine cusp tip;**Inter molar perpendicular (IMW per) =a**: The perpendicular distance from the midpoint of intermolar width to the labial surface of maxillary central incisors. This is equivalent to the major axis of Ramanujan's equation for an ellipse.

**Figure 3:**
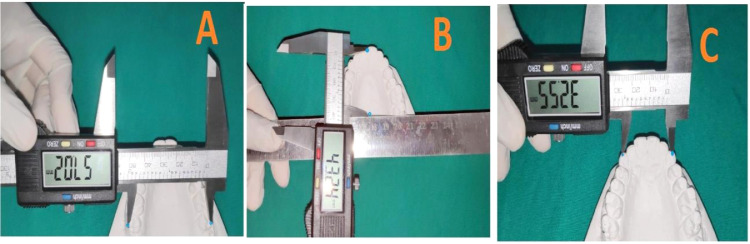
**Measurement of Intermolar width(IMW**); B- Measurement of Intermolar width perpendicular (IMW per); C- Measurement of Intercanine width(ICW). Measurements were done directly on the models with a digital Vernier calipers *(iGaging ®, LA, California, USA)*.

**Figure 4:**
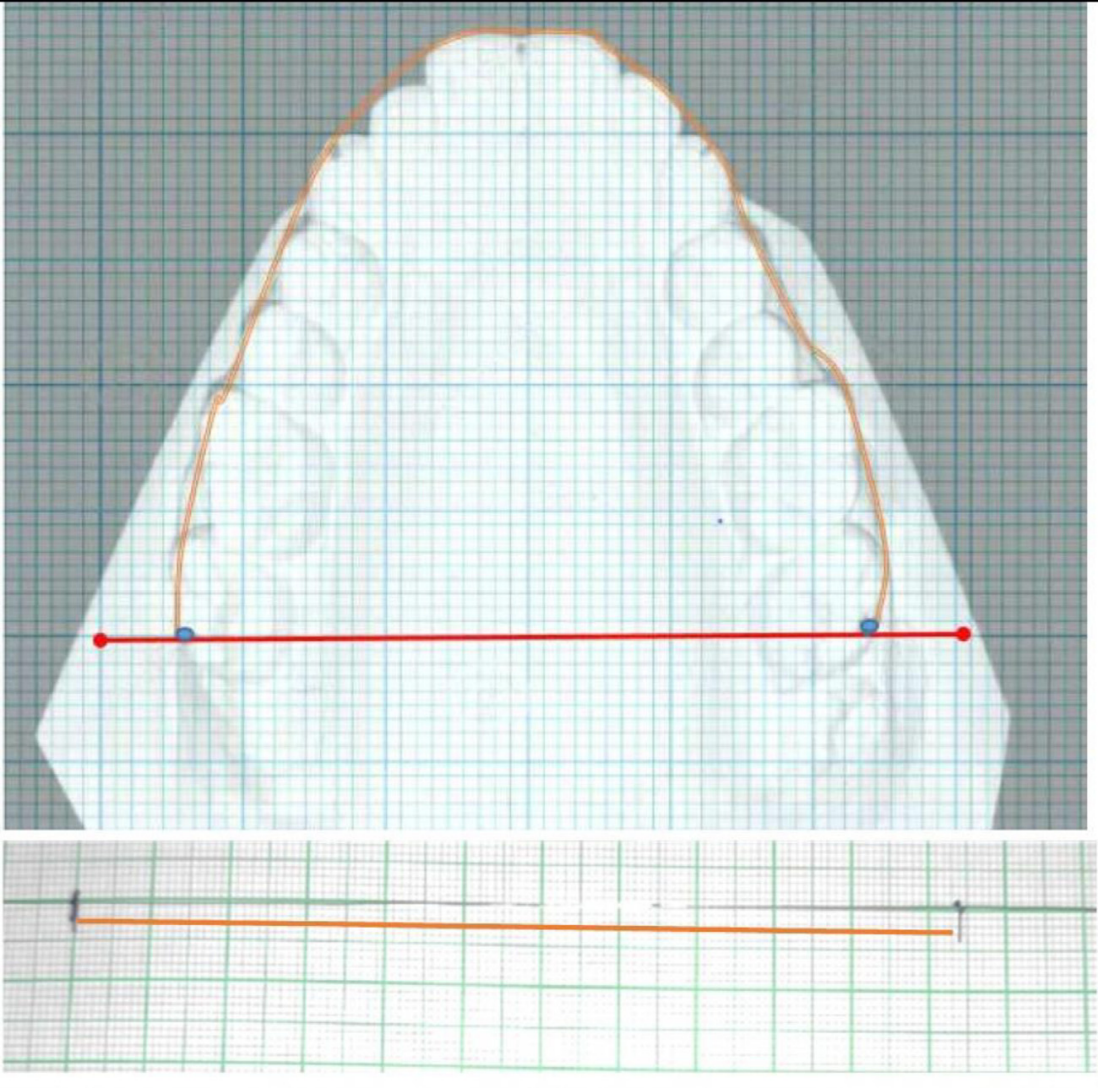
**Measured arch perimeter(MP)** The arch-perimeter was directly measured on the plaster models from the vertical line marked on the distobuccal cusp of maxillary second molars with a 0.010inch stainless steel ligature wire that contacted the buccal surface of each posterior tooth and labial surfaces of each anterior tooth. The wire was marked at distobuccal cusps with a marking pencil, and then it was straightened and laid flat on a graph paper.

**Figure 5:**
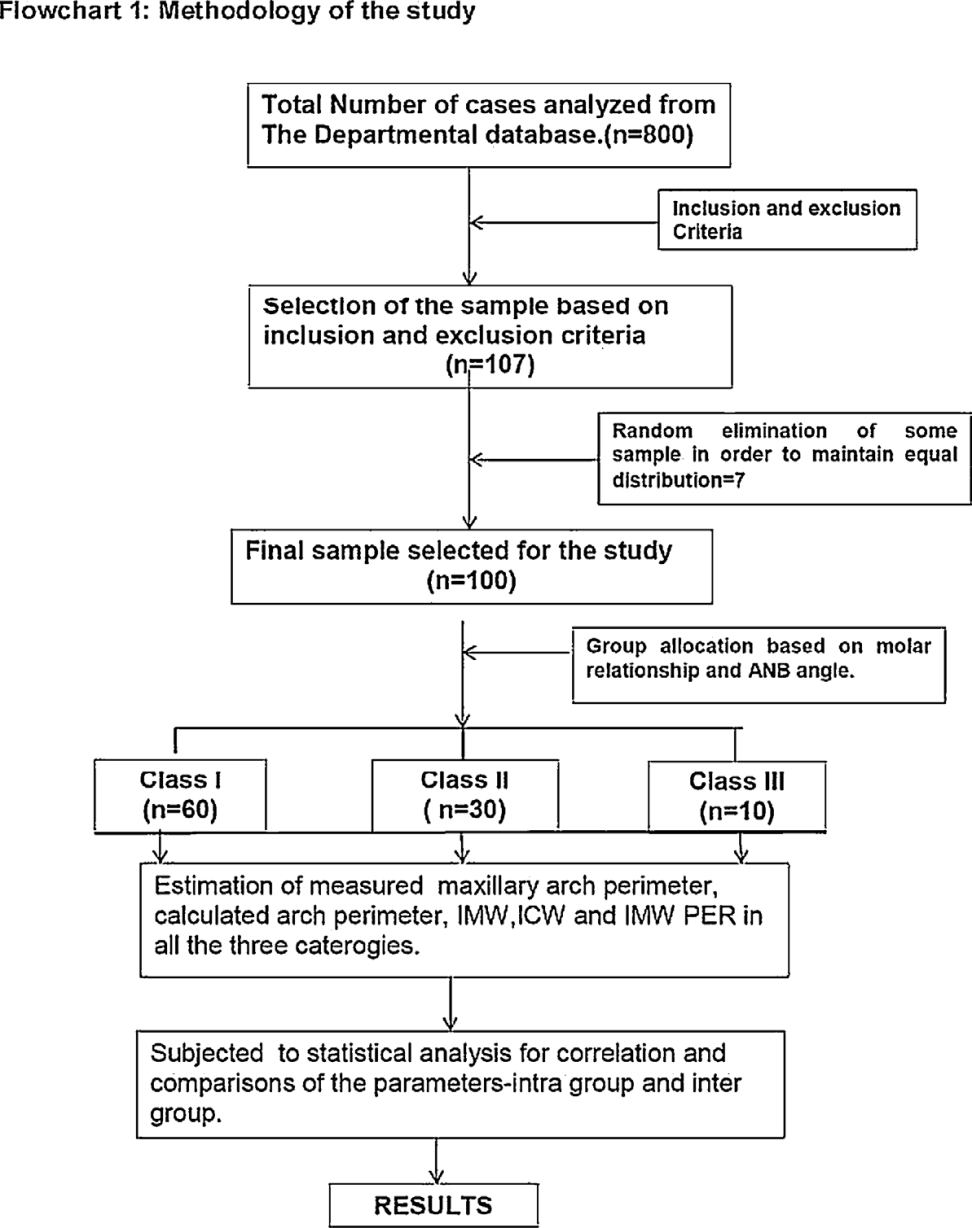
Flow chart of the procedure of collection of the data.

The data set includes 5 tables, 5 figures and three graphs. describes the Ramanujan's equation of Ellipse model (Figure 1) the Parameters defined in the collection of data (Figure 2), procedures to measure the parameters (Figure 3, Figure 4) and Figure 5 describes the flow chart of the procedure. It includes raw data provided in the supplementary files. The analyzed tables include the results of the Dahlberg's error estimation (Table 1), Test of Normality (Table 2), The marginal difference Correlation between MP and CP (Table 3), comparison of variables between different groups (Table 4) and pair wise comparison of the groups for the variables (Table 5). The three graphs included in the data represented the correlation between the MP and CP in all the three groups (Graph I, Graph II, Graph III). The algorithm steps in arriving at the statistical formulae for direct entry to the complex mathematical equation is provided in the supplemental files.

**Table 1:** Dahlberg’s error.

IMW	IMW PER	MP	ICW
0.058 or 5.75%	0.077 or 7.71%	0.280 or 27.95%	0.028 or 2.79%

**Table 2:** Test of normality.

	Class	Shapiro-Wilk		
		Statistic	df	p-value
**IMW**	**1**	0.92	60	0.001*
	**2**	0.92	30	0.02*
	**3**	0.82	10	0.03*
**IMW PER**	**1**	0.79	60	<0.001*
	**2**	0.99	30	0.98(NS)
	**3**	0.91	10	0.26(NS)
**MP**	**1**	0.94	60	0.004*
	**2**	0.93	30	0.05*
	**3**	0.75	10	0.003*
**CP**	**1**	0.73	60	<0.001*
	**2**	0.97	30	0.59(NS)
	**3**	0.93	10	0.47(NS)
**ICW**	**1**	0.63	60	<0.001*
	**2**	0.95	30	0.17(NS)
	**3**	0.87	10	0.09(NS)

*p<0.05 Statistically Significant, p>0.05 Non Significant, NS.

**Table 3:** Correlation between MP and CP class I, class III, and class III groups. Test statistic- spearman correlation test.

Categories	n	MP	CP	Difference in median values *(mm)*	Marginal difference in relation to MP %	Correlation coefficient ‘S_r_’	‘P’ value
		Median *(mm)*	Median *(mm)*				
**Class I**	**n=60**	121.96	122.20	0.24	0.19%	0.84	<0.001**
**Class II**	**n=30**	125.28	126.46	1.18	0.09%	0.52	0.003**^s^
**Class III**	**n=10**	120.55	118.09	-2.46	2.04%	0.49	0.15^NS^
**Overall percentage error**	**n=100**	122.64	124.13	1.49	1.21%	0.6809	<0.0001**

**p<0.01 -very Significant; *p<0.05 –Significant; p>0.05, NS- Non Significant.

***MP -****Measured arch perimeter****: CP - C****alculated perimeter. All measurements are in millimeters (mm)*.

**Table 4:** Comparison of study variables between the class of malocclusion (All measurements in mm).

Class		n	Mean (SD)	Range	Median(Q1-Q3)	Kruskal Wallis test	
					Chi Square value	p-value	
**IMW**	**Class 1**	60	56.78 (3.77)	40.48- 63.48	57.22(54.56- 59.43)	23.04	<0.001*
	**Class 2**	30	59.51 (3.00)	53.72- 63.44	59.86(56.28- 62.34)		
	**Class 3**	10	62.66 (3.60)	55.56- 67.40	63.78(61.09- 64.37)		
**IMW PER**	**Class 1**	60	47.24 (3.98)	25.78- 54.82	47.45(45.77- 49.29)	20.38	<0.001*
	**Class 2**	30	48.30 (2.43)	43.32- 54.48	48.20(46.56- 50.13)		
	**Class 3**	10	42.89 (1.85)	40.68- 45.66	42.69(41.17- 44.99)		
**MP**	**Class 1**	60	121.86 (6.06)	98.69- 139.27	121.96(119.25- 124.70)	12.05	0.002*
	**Class 2**	30	124.97 (4.44)	110.54- 131.74	125.28(123.14- 127.94)		
	**Class 3**	10	121.60 (4.13)	117.84- 132.38	120.55(119.05- 122.72)		
**CP**	**Class 1**	60	121.60 (8.43)	72.68- 135.66	122.20(119.47- 125.83)	14.68	0.001*
	**Class 2**	30	125.57 (5.17)	114.06- 133.73	126.46(121.31- 129.94)		
	**Class 3**	10	117.66 (5.06)	109.95- 124.51	118.09(114.12- 122.89)		
**ICW**	**Class 1**	60	35.85 (2.95)	17.22- 40.36	36.16(35.11- 37.32)	35.83	<0.001*
	**Class 2**	30	34.92 (1.15)	32.18- 36.83	35.19(34.15- 35.77)		
	**Class 3**	10	41.57 (2.26)	38.99 - 44.77	40.84(39.41- 44.40)		
**ICW/IMW**	**Class 1**	60	0.62 (0.052)	0.42-0.73	0.62(0.59-0.66)	23.92	<0.001*
	**Class 2**	30	0.58(0.038)	0.52-0.56	0.59(0.55-0.61)		
	**Class 3**	10	0.66(0.03)	0.60-0.72	0.66(0.63-0.70)		
**IMW/IMW per**	**Class 1**	60	1.19 (0.10)	0.84-1.42	1.19(1.13-1.25)	46.54	<0.001*
	**Class 2**	30	1.09(0.05)	0.98-1.16	1.09(1.03-1.14)		
	**Class 3**	10	1.46(0.07)	1.33-1.57	1.45(1.40-1.52)		

*p<0.05 Statistically Significant, p>0.05 Non Significant, NS.

**Graph I:**
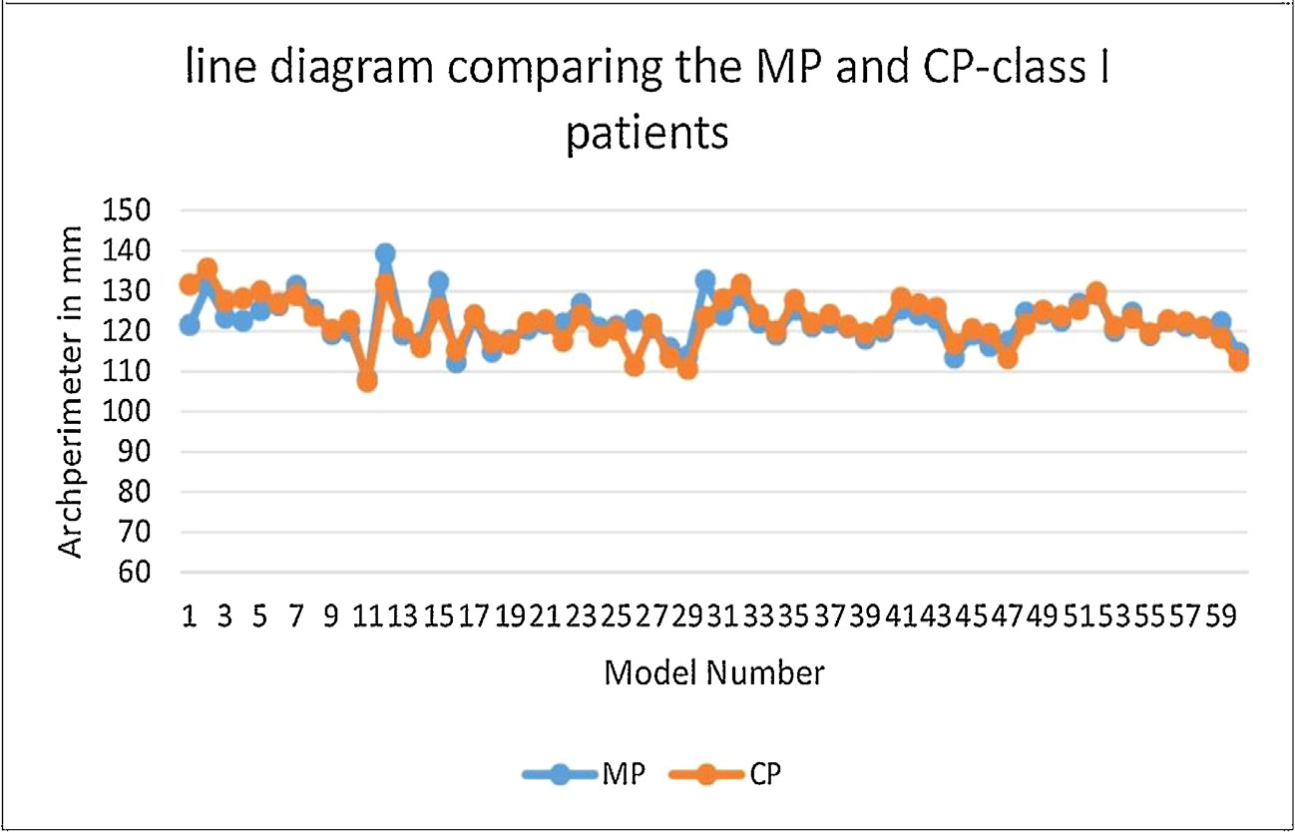
correlation between Measured(MP) and calculated (CP)arch perimeter - class I.

**Graph II:**
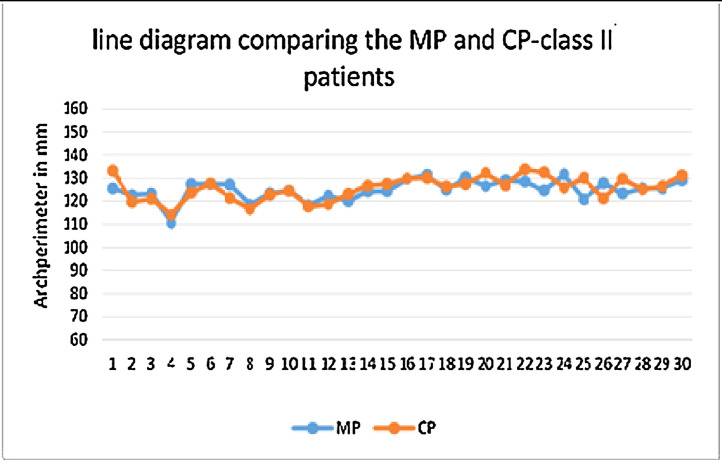
correlation between Measured(MP) and calculated (CP)arch perimeter - class II.

**Graph III:**
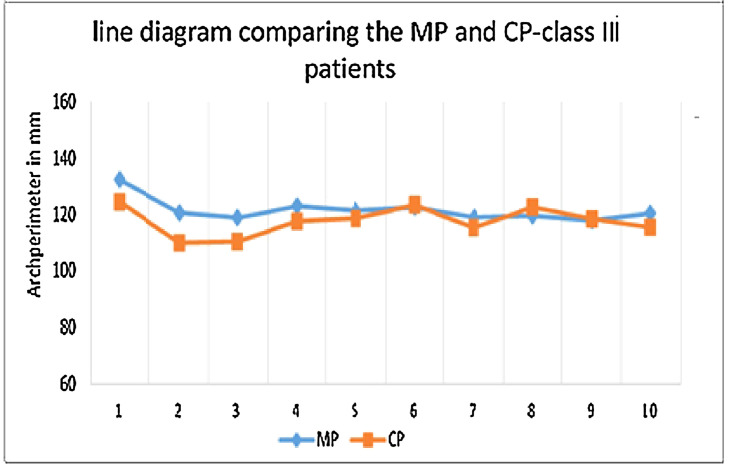
correlation between Measured(MP) and calculated (CP)arch perimeter - class III.

## 2 Experimental design, materials and methods

Samples were taken from plaster models of the maxillary arch of the patients aged 15-30 years, of three different sagittal malocclusion categories. The data was collected from the measurements made on the models of three different categories of malocclusion, Class I-n(60); Class II-n(30); and Class III-n(10) based on the prevalence in the population [9]. The well-aligned dentitions with TSALD discrepancy of less than 3 mm and from the patients who had received no prior orthodontic treatment with fully erupted second molars. The cephalometric criteria included ANB values of ±4 with no vertical (FMA angle<30 degrees) and transverse skeletal dysplasia [10]. The Institutional Ethical committee approval for this data collection was obtained. The procedures were explained to the patient and after obtaining a written consent. All the procedures followed herewith for the preparation of models are established standard safety protocols.

An ellipse based on Ramanujan's equation for calculation of the perimeter was fitted to the Maxillary arch [2] (Figure 1). The parameters for collection of the data consist of measured and calculated data (Figure 2). All the primary linear measurements, such as IMW, IMW per, ICW, were made with digital Vernier calipers *(iGaging ®, LA, California, USA)*. The data for the Measured arch perimeter (MP) according to the procedure mentioned [2] (Figure 3). The Calculated arch perimeter(CP) was obtained from the measured data after inserting them into Ramanujan's equation for calculation of the perimeter of an ellipse (Figure 2). The semi-major axis ’a’ of the ellipse is equivalent to the IMW per. Clinically this measurement indicates the Incisor proclination. The input for the ‘b’ is equivalent to half the IMW measured. The formula for Ramanujan's equation for ellipse was coded in excel sheet by using an Algorithm for expression of the model.

$$\begin{array}{c} \{=(3.14159n65358979*(\mathrm{An}\pm \mathrm{Bn}))*(1\pm ((3*((\left(\mathrm{An}*\mathrm{An}\right)\pm \left(\mathrm{Bn}*\mathrm{Bn}\right)-n*\mathrm{An}*\mathrm{Bn}))/(\left(\mathrm{An}*\mathrm{An}\right)\pm \\ \left(\mathrm{Bn}*\mathrm{Bn}\right)\pm \left(n*\mathrm{An}*\mathrm{Bn}\right))))/(10-(\mathrm{SQRT}((4-3*((\left(\mathrm{An}*\mathrm{An}\right)\pm \left(\mathrm{Bn}*\mathrm{Bn}\right)\left(n*\mathrm{An}*\mathrm{Bn}\right))/(\left(\mathrm{An}*\mathrm{An}\right)\\ \pm \left(\mathrm{Bn}*\mathrm{Bn}\right)\pm \left(n*\mathrm{An}*\mathrm{Bn}\right)))))))\}. \end{array}$$

The values were directly and automatically derived after entering the values for ‘a' and 'b'. The automated values of Ramanujan's equation for ellipse was 99.99 % accurate when verified against stepwise calculation for the equation on ten randomly selected models. The primary researcher(JSYP) performed all the measurements and data acquisition. The reproducibility of the measurements recorded was evaluated after 2 weeks by Dahlberg's formula test [11] (Table 1) which showed a matching of above 90 percent for all the parameters. The data obtained from the measurements were entered as quantitative measurements in millimeters (mm) in the excel data sheet (Microsoft 2007) for all the three groups separately.

## 3 Data analysis

The data collected was entered into Microsoft excel spreadsheet and analyzed using IBM SPSS Statistics, Version 22(Armonk, NY: IBM Corp). Descriptive data were presented in the form of mean, median, standard deviation and quartiles for continuous variables. Non-parametric tests were applied as the data were of not a normal distribution (Table 2). Spearman's Correlation test was used to test the correlation between the variables (Table 3) [Graph 1,2,3]. Comparison of the data variables between three groups was done using kruskall Wallis test followed by Mann whitney U test as post hoc test (Table 4 and Table 5). The P value < 0.05 was considered as statistically significant.

**Table 5:** Pairwise comparison of study variables between the class of malocclusion.

	Class 1 vs Class 2		Class 1 vs Class 3		Class 2 vs Class 3	
	U Statistic	p-value	U Statistic	p-value	U Statistic	p-value
**IMW**	513	0.001*	74.5	<0.001*	54.5	0.003*
**IMW PER**	781.5	0.31(NS)	57	<0.001*	10	<0.001*
**MP**	534.5	0.002*	253	0.43(NS)	66	0.009*
**CP**	593.5	0.009*	164	0.02*	39.5	0.001*
**ICW**	500	0.001*	4	<0.001*	0	<0.001*
**ICW/IMW**	422	< .00001*	186	< .00001*	29	< .00001*
**IM/IMper**	305	< .00001*	13	< .00001*	0	< .00001*


	Class 1 vs Class 2		Class 1 vs Class 3		Class 2 vs Class 3	
	U Statistic	p-value	U Statistic	p-value	U Statistic	p-value
**IMW**	513	0.001*	74.5	<0.001*	54.5	0.003*
**IMW PER**	781.5	0.31(NS)	57	<0.001*	10	<0.001*
**MP**	534.5	0.002*	253	0.43(NS)	66	0.009*
**CP**	593.5	0.009*	164	0.02*	39.5	0.001*
**ICW**	500	0.001*	4	<0.001*	0	<0.001*

Mann Whitney U test.

*p<0.05 Statistically Significant, p>0.05 Non Significant, NS.

## Ethics statement

Informed consent was obtained for collection of data from the human subjects; All the procedures are established safety norms without any harm to the patient.

## Declaration of Competing Interest

The authors declare that they have no known competing financial interests or personal relationships which have, or could be perceived to have, influenced the work reported in this article.

## Appendix Supplementary materials

Image, application 1

Image, application 1
