# Supplementary material for: Data Set For Computation Of Maxillary Arch Perimeter With Ramanujan's Equation For Ellipse In Different Skeletal Malocclusions
Source: Data Brief. 2020 Jul 25;32:106079. doi: 10.1016/j.dib.2020.106079 (PMC7403886; doi:10.1016/j.dib.2020.106079)
Supplement: Supplementary file 2 [file mmc2.docx]

**Table 1.Dahlberg’s Error**

| **IMW** | **IMW PER** | **MP** | **ICW** |
| --- | --- | --- | --- |
| 0.058 or 5.75% | 0.077 or 7.71% | 0.280 or 27.95% | 0.028 or 2.79% |
